# Near pan-alphavirus neutralization by an antibody-nanobody bispecific

**DOI:** 10.1080/19420862.2026.2731281

**Published:** 2026-09-23

**Authors:** Sergei Pletnev, Christina L. Gardner, Matthew S. Sutton, Baoshan Zhang, Victoria Callahan, Courtney Green, Nicholas C. Morano, Tatsiana Bylund, Megan M. Dunagan, Yaroslav Tsybovsky, Ellen R. Goldman, Lawrence Shapiro, Crystal W. Burke, Julie M. Fox, Mario Roederer, Peter D. Kwong, Tongqing Zhou

**Affiliations:** a Vaccine Research Center, National Institute of Allergy and Infectious Diseases, National Institutes of Health, Bethesda, MD, USA; b Virology Division, U.S. Army Medical Research Institute of Infectious Diseases, MD, USA; c Chenega Professional & Technical Services, Chesapeake, VA, USA; d Emerging Virus Immunity Unit, Laboratory of Viral Diseases, National Institute of Allergy and Infectious Diseases, National Institutes of Health, Bethesda, MD, USA; e Aaron Diamond AIDS Research Center, Department of Medicine, Vagelos College of Physicians and Surgeons, Columbia University, New York, NY, USA; f Department of Biochemistry and Molecular Biophysics, Columbia University, New York, NY, USA; g Zuckerman Mind Brain Behavior Institute, Columbia University, New York, NY, USA; h Electron Microscopy Laboratory, Cancer Research Technology Program, Frederick National Laboratory for Cancer Research, Frederick, MD, USA; i Center for Biomolecular Science and Engineering, U.S. Naval Research Laboratory, Washington, DC, USA

**Keywords:** Antibody improvement, antibody-nanobody bispecific, bispecific antibody, SKT05 antibody, V2C3 nanobody

## Abstract

Alphaviruses represent a global concern, with some such as chikungunya virus currently infecting thousands in China and others such as Venezuelan equine encephalitis virus inducing neurological symptoms in 4–14% of infected individuals. A single antibody capable of protecting from all human-infecting alphaviruses, by achieving sufficient potency against each virus individually, may have high utility, as only a single therapeutic would then need to be stockpiled against this entire class of emerging pathogens. We previously identified an antibody, SKT05, with broad encephalitic alphavirus recognition, and we also identified nanobodies capable of neutralizing both arthritogenic and encephalitic alphaviruses. Here, we employ structure-based design to engineer a SKT05-nanobody bispecific, which has near pan-neutralization of alphaviruses that infect humans. Twenty-three designed SKT05-nanobody bispecifics broadly neutralized pseudotyped viruses expressing envelope proteins of encephalitic alphaviruses. With 10 of these bispecifics, we further assessed live arthritogenic alphavirus neutralization for Mayaro, O’nyong’nyong, Ross River, and Una viruses, observing potent neutralization for all of them with four bispecifics. The best of these four, V2C3-30-SKT05H, comprising the V2C3 nanobody genetically fused through a 30-amino acid linker to the heavy chain of SKT05, potently neutralized 9 of 11 tested alphaviruses that were phylogenetically proximal to all 26 alphaviruses that infect and cause symptoms in humans. Further, therapeutic administration of V2C3-30-SKT05H reduced viral loads in chikungunya virus-infected mice. Electron microscopy revealed that V2C3-30-SKT05H induced disorder of E1E2 spikes on the viral surface, suggesting E1E2 disassembly as a potential neutralization mechanism. Collectively, these results demonstrate near pan-neutralization of human-infecting alphaviruses by an antibody-nanobody bispecific.

## Introduction

Alphaviruses are enveloped, positive-sense, single-stranded RNA viruses of the Togaviridae family with substantial impact on human and animal health primarily through mosquito transmission. In humans, alphavirus infection can result in either arthritis or encephalitis, depending on the infecting alphavirus. Arthritogenic alphaviruses, including Ross River virus (RRV) and chikungunya virus (CHIKV), cause epidemics that are increasing in both range and frequency, particularly in several regions of Africa and Asia, including a 2025 CHIKV outbreak that has infected over 7,000 individuals in the Guangdong Province of China.^1^ These viruses can cause acute and chronic musculoskeletal disease, which while rarely fatal, can result in persistent joint pain often lasting for months to years after infection.^2–4^ In contrast, symptoms from encephalitic alphavirus infection, including western, eastern, and Venezuelan equine encephalitis virus (WEEV, EEEV, and VEEV, respectively), can vary from asymptomatic or acute febrile illness to severe neurological impairment and death.^5,6^ EEEV is the most virulent, with a human fatality rate of 30–75% in symptomatic individuals presenting with neuro-invasive disease.^6–8^ VEEV, while the least lethal, may be the most significant in terms of morbidity.^9–12^ Outbreaks of VEEV can become self-sustaining, reaching levels sufficient for domestic/urban transmission between humans.^5,13^ Most infections with VEEV are apparent and present abruptly with debilitating flu-like symptoms.^8,12,14^

Multiple vaccine and therapeutic candidates have been explored to combat alphavirus infections, but only a few have been approved in the United States.^15–22^ Though transmission is primarily by mosquitos, all three equine encephalitis viruses (EEVs) can be transmitted via aerosol exposure.^12,23^ Accordingly, the EEVs are considered National Institute of Allergy and Infectious Diseases Category B priority pathogens, with EEEV and VEEV being U.S. Department of Agriculture / Centers for Disease Control and Prevention Select Agents due to their potential as bioterrorism agents.^24,25^ Investigational vaccines against each EEV have been used by the US military since the 1970s for at-risk laboratory and military personnel, but have resulted in poor immunogenicity, limited longevity, and substantial side effects.^20,26,27^ A trivalent vaccine consisting of individual WEEV, EEEV, and VEEV (WEVEEV) virus-like particles (VLPs) demonstrated good immunogenicity in nonhuman primates (NHP) and protected them against aerosol challenge with all three EEVs^19^; analysis of vaccinated NHPs identified an antibody, SKT05, that protected against VEEV, CHIKV, and RRV, suggesting broad therapeutic protection against alphaviruses might be possible.^28^

In addition to antibodies that protect from alphavirus infection,^29–34^ variable heavy domains of heavy-chain-only antibodies (nanobodies) have been identified with broad alphavirus neutralization, including V2B3, V2C3, and V3A8f, from immunized llamas,^35^ and we recently determined their structures in complex with VEEV VLPs.^36^ No single antibody or nanobody capable of pan-alphavirus neutralization, however, has been identified.

We previously found bispecific antibody-nanobody-based molecules could provide increased breadth and potency of neutralization against HIV-1, with promising half-lives in human FcRn knock-in mice and manufacturing productivity higher than the parent antibody.^37,38^ We thus wondered: Could an antibody-nanobody bispecific provide pan-alphavirus protection? To investigate, we designed bispecific antibody-nanobodies, by analyzing previously reported structures of antibody SKT05 and nanobodies V2B3, V2C3, and V3A8f in complex with alphavirus VLPs^28^ and determined appropriate termini that are not sterically occluded upon antibody binding to VLP and linker lengths to genetically fuse each nanobody to SKT05. We assessed neutralization for 23 bispecific antibodies on pseudoviruses expressing the E2 and E1 of WEEV, EEEV, or VEEV from which we selected 10 bispecifics to evaluate neutralization on live arthritogenic alphaviruses. We also assessed the top 2 bispecifics on a panel of 11 live arthritogenic and encephalitic alphaviruses, and the best bispecific in an *in vivo* murine CHIKV model. Lastly, we used electron microscopy to assess VEEV VLPs in the presence of an antibody-nanobody bispecific to provide insight into the mechanism of virus neutralization. Overall, we designed and characterized anti-alphavirus antibody-nanobody bispecifics. The best, V2C3-30-SKT05H, induced VLP trimer disorder, neutralized all 11 tested human-infecting arthritogenic and encephalitic alphaviruses, and showed *in vivo* efficacy against CHIKV in a therapeutic murine model.

## Results

### Design of nanobody-SKT05 bispecifics

**Figure 1.**
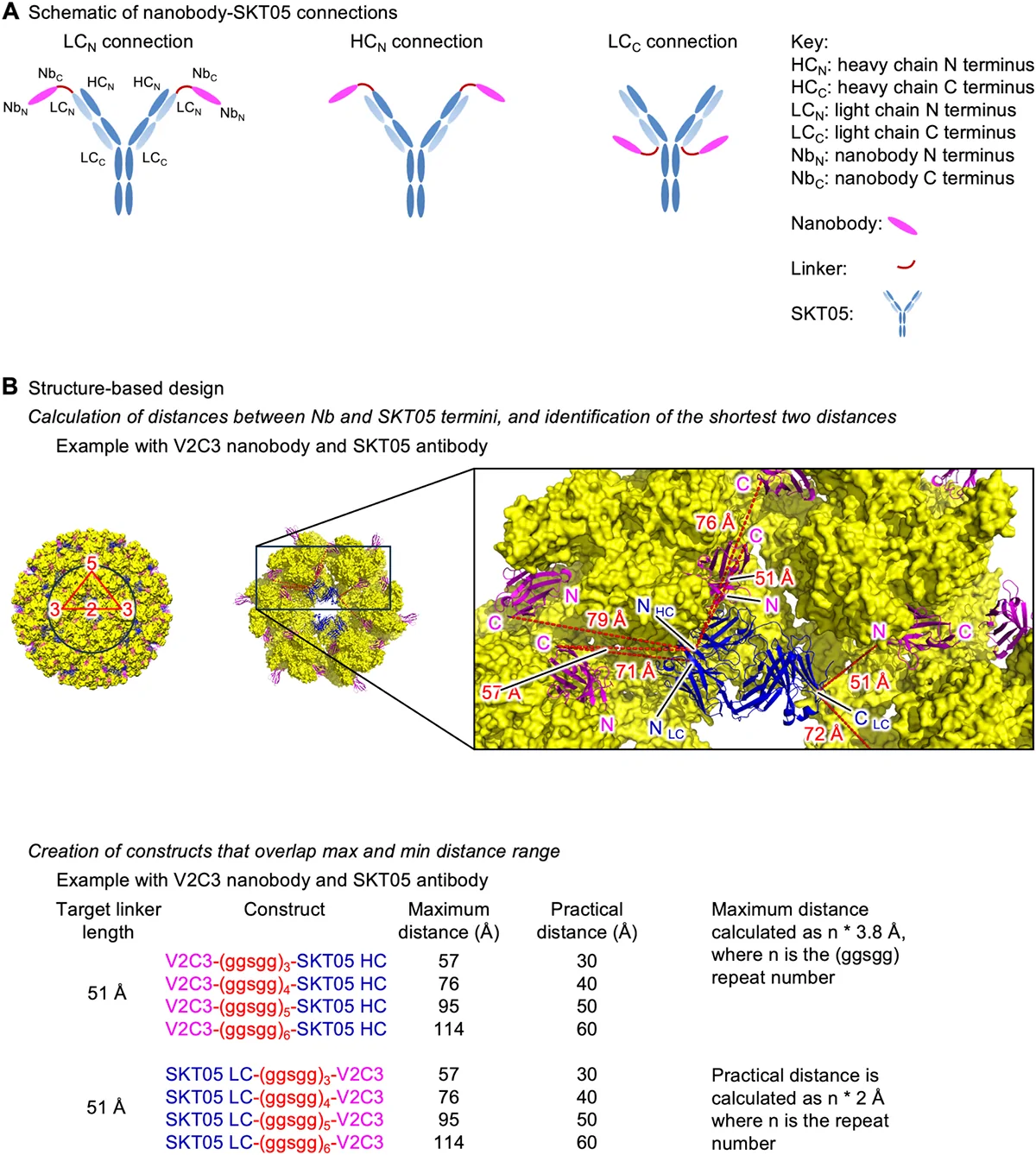
Design of nanobody-SKT05 bispecifics. (A) Schematics are shown for connections (red) between nanobodies (magenta) and SKT05 antibody (blue). Light chain and heavy chain termini and also nanobody termini are indicated by subscript N for N terminus and C for C terminus. (B) Design and example with V2C3 nanobody and SKT05 antibody are shown. Constructs and distances are shown for V2C3-SKT05 bispecifics. The (ggsgg)_n_ linker peptide is gly-gly-ser-gly-gly, repeated n times. The VLP and all zoom-in panels are oriented the same way, with the icosahedral two-fold axes aligned with the cartesian x-, y-, and z-axes. Left, major icosahedral symmetry elements displayed on the entire VLP. Central, the six spikes around the icosahedral 2-fold.

To design nanobody-SKT05 bispecifics, we first modeled the missing SKT05 CL and CH1 domains into the published structure of the broadly reactive SKT05 antibody in complex with VEEV VLP (PDB ID: 8EEU).^28^ The resultant model revealed the SKT05-N termini for both heavy and light chains as well as the C terminus of the light chain to be unencumbered by antibody-VLP interaction and free for linkage to a nanobody (Figure 1A). Meanwhile, we recently determined the structures of several nanobodies in complex with VEEV VLP^36^ delineating their binding to the VEEV VLP. With structures of nanobodies and SKT05 in complex with VEEV VLP, we could identify appropriate sites and distances for linkage. We delineated the shortest two distances to link nanobodies and termini on SKT05 antibody; for each of these distances, we created a range of linkers with tandem repeats of the GGSGG pentapeptide sequence, which spanned the shortest maximal distance (estimated at 3 Å per amino acid) and for the longest practical distance (estimated at 2 Å per amino acid) (Figure 1B). The choice of the 2–3 Å/residue range was intended to eliminate excessively long linkers (generated if less than 2 Å/residue distance is used) and to avoid approaching the energetically unfavorable fully extended polypeptide chain conformation (3.8 Å/residue).

The V2B3 nanobody bound to the interface between three molecules on the VEEV VLP: E1 and E2 of one trimeric spike as well as E1 from an adjacent trimeric spike (PDB ID 9NRX), effectively locking the trimers of the VLP together. We measured all distances less than 90 Å, between the N terminus of nanobody and C terminus of SKT05 light chain, which could therefore be genetically linked by a construct starting with antibody light chain at its N terminus and joined by a linker to the N terminus of the V2B3 nanobody (Fig. S1). In total, five distances of under 90 Å were measured, of which the two shortest were 46 and 58 Å (Fig. S1B), which linked the C terminus of nanobody V2B3 with the heavy chain of SKT05 (46 Å) or with the light chain of SKT05 (58 Å). With these target distances in mind, we designed and made three constructs for V2B3-(ggsgg)_n_-SKT05 HC (with linkers ranging from *n* = 3–5) and three constructs for V2B3-(ggsgg)_n_-SKT05 LC (with linkers ranging from *n* = 4–6), covering target linker length distances of 46 and 58 Å (Fig. S1C).

By using a similar approach, we also designed V2C3-SKT05 bispecifics, based on the complex structure of nanobody V2C3 with VEEV VLP (PDB ID 9NRY), which bound to an epitope that overlapped the fusion loop at the interface of the E1 and E2 spike subunits. We generated a composite model of V2C3 and SKT05 on the VEEV VLP (Fig. S2A), checked for the absence of spatial overlaps between non-bonded atoms of 0.4 Å or greater between V2C3 and SKT05, and measured all distances less than 90 Å between the V2C3 C terminus and the SKT05 heavy and light chain N termini, as well as between the V2C3 N terminus and the SKT05 light chain C terminus (Fig. S2B). Of a total of seven distances that were under 90 Å, the two shortest were both 51 Å, occurring: 1) between V2C3 C terminus and SKT05 heavy chain N terminus, comprising an antibody-nanobody linkage site that can be used to fuse V2C3 with SKT05 heavy chain via flexible (ggsgg)_n_ linker; and 2) between SKT05 light chain C terminus and V2C3 N terminus, comprising a linkage site that can be used to engineer a reverse construct in which SKT05 light chain is genetically linked with V2C3 via (ggsgg)_n_ linker. Overall, we made four constructs for V2C3-(ggsgg)_n_-SKT05 HC and four constructs for SKT05 LC-(ggsgg)_n_-V2C3 with linkers ranging from *n* = 3–6, covering the target linker length distance of 51 Å (Fig. S2C).

Similarly, we used the structure of V3A8f nanobody in complex with VEEV VLP (PDB ID 9NRZ) to design V3A8f bispecifics with SKT05 antibody. V3A8f binds to a site that overlaps the fusion loop at the E1 and E2 interfaces. We generated a composite model of V3A8f and SKT05 on VEEV VLP and checked for the spatial overlaps between V3A8f and SKT05 (Fig. S3A). Although V3A8f does not overlap with SKT05, nanobody molecules bound to the VEEV VLP form a few 2.1 Å contacts with the antibodies (Fig. S3B). To identify potential linkers between nanobody and antibody, we measured all distances less than 90 Å between the V3A8f C terminus and SKT05 heavy and light chain N termini, as well as between V3A8f N terminus and SKT05 light chain C terminus, selected the two shortest (43 Å between V3A8f C-terminus and SKT05 heavy chain N terminus and 25 Å between SKT05 light chain C terminus and V3A8f N terminus), and designed nine constructs (four for V3A8f-(ggsgg)_n_-SKT05 HC (with linkers ranging from *n* = 3–6) and five for SKT05 LC-(ggsgg)_n_-V3A8f (with linkers ranging from *n* = 2–6) covering target linker lengths (Fig. S3C).

In sum, we designed 23 SKT05-nanobody constructs using knowledge of the spatial disposition of nanobodies and SKT05 on VEEV VLP; this was instrumental in identifying permissible combinations of components and linker lengths, thereby reducing the number of combinations from 81 to 23 practical designs.

### Neutralization assessment of designed SKT05-nanobody bispecifics

**Figure 2.**
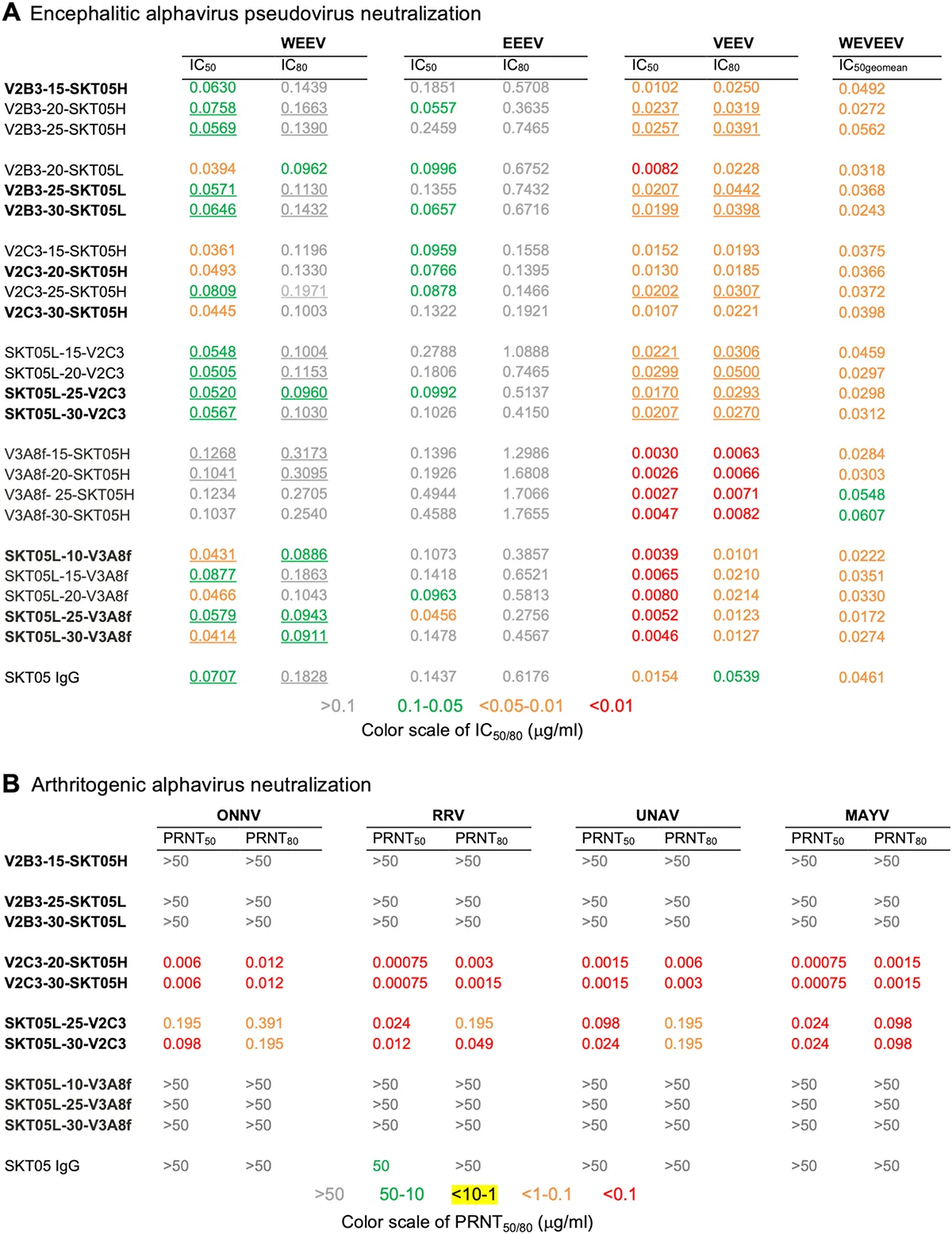
Encephalitic pseudovirus and arthritogenic live virus neutralization by designed SKT05-nanobody bispecifics. (A) Pseudovirus encephalitic alphavirus (WEEV, EEEV, and VEEV) neutralization. IC_50/80_ is provided and colored as indicated. Underlined values were remeasured because initial concentrations were not diluted sufficiently; initial values versus repeat values are shown in fig. S11. WEVEEV IC_50_geomean is provided to demonstrate the overall effectiveness of the constructs. Constructs shown in bold were selected for further testing of live-virus neutralization of the arthritogenic alphaviruses. (B) live virus neutralization of various arthritogenic alphaviruses (ONNV, RRV, UNAV, and MAYV). PRNT_50/80_ is provided and colored as indicated; we provide both PRNT50 and PRNT80 to inform readers of the shape of the neutralization curve, with full neutralization data provided in supplemental fig. S5.

Having designed 23 SKT05-nanobody constructs, we next expressed and tested their binding and pseudovirus neutralization capacity. As reference, we also assessed the parent SKT05 antibody using the same methods. Starting with the encephalitic alphavirus (EEEV, VEEV and WEEV), we assessed the binding of each of the 23 bispecifics and SKT05 to VEEV, EEEV, and WEEV VLPs (Fig. S4, Figure S5A). Notably, all of the 23 bispecifics and SKT05 bound to VLPs of VEEV, EEEV, and WEEV. We also tested the ability of each of the 23 bispecifics and SKT05 to neutralize encephalitic alphavirus Env-pseudotyped viruses (Figure 2A, Figure S5B). The neutralization for VEEV was best, less potent for WEEV, and substantially weaker for EEEV. In general, all combinations showed similar binding and neutralization, except for V3A8f linked to the heavy chain of SKT05 and SKT05 itself, for which EEEV neutralization was less robust.

We then selected 10 of the SKT05-nanobody constructs for further testing of live-virus neutralization of the arthritogenic alphaviruses, o’nyong’nyong virus (ONNV), RRV, Una virus (UNAV), and Mayaro virus (MAYV) (Figure 2B, Figure S6). Parent SKT05 antibody was included in this assay as a reference. In contrast to the broad encephalitic pseudovirus neutralization, only four bispecifics showed neutralization activity. These were SKT05L-30-V2C3, SKT05L-25-V2C3, V2C3-20-SKT05H, and V2C3-30-SKT05H. Of these four, the two with nanobodies linked to SKT05H had the largest reduction in infection by plaque reduction neutralization test (PRNT) against the alphaviruses tested. Parent SKT05 also did not show neutralization activity except for RRV for which a borderline neutralization was detected.

### Neutralization of diverse alphaviruses by lead bispecifics

**Figure 3.**
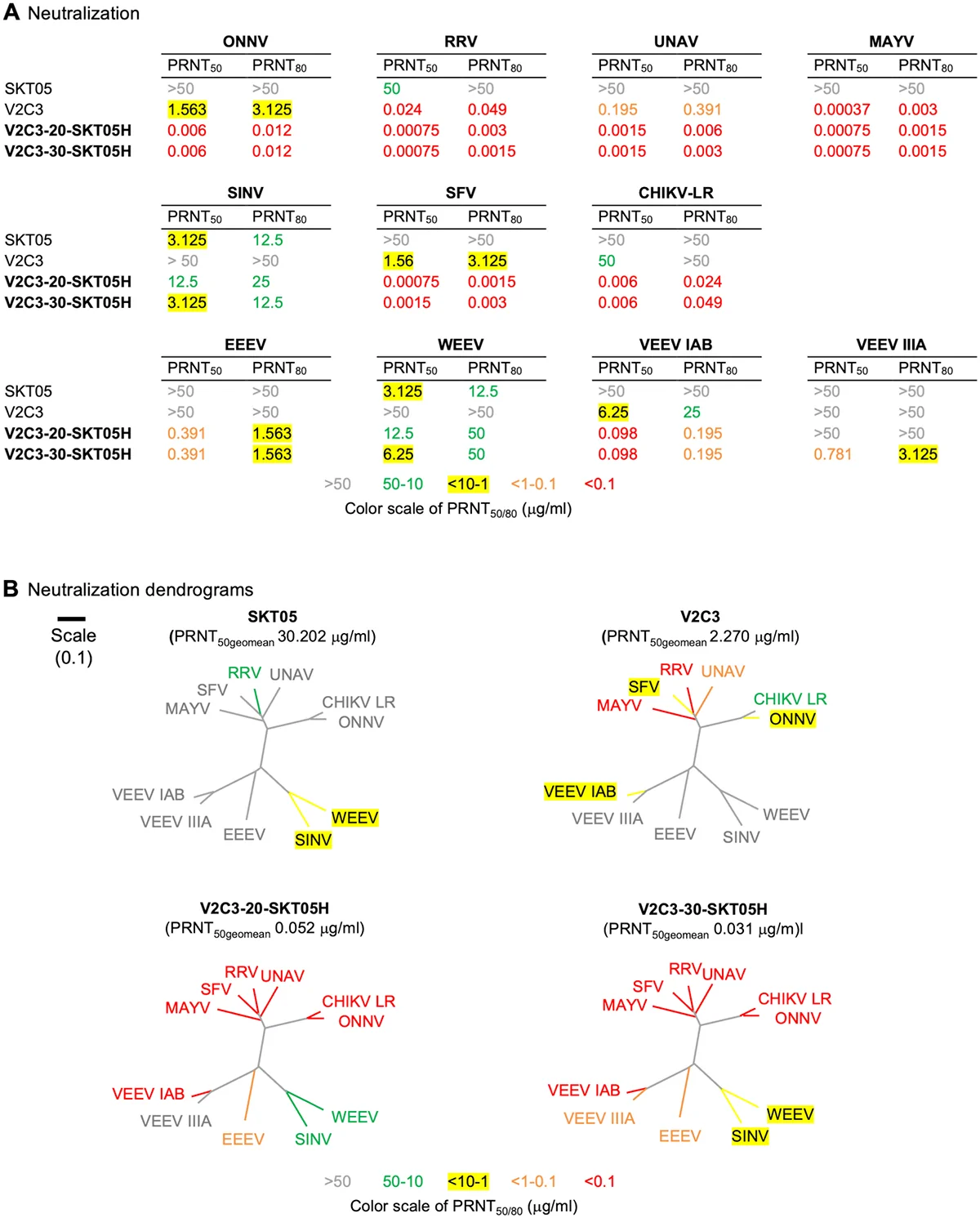
Live virus assessment of two lead bispecific reveals V2C3-30-SKT05H to pan-neutralize diverse alphaviruses. (A) Neutralization of arthritogenic (ONNV, RRV, UNAV, MAYV, SINV, SFV, and CHIKV-LR) and encephalitic (EEEV, WEEV, VEEV IAB, and VEEV IIIA) alphaviruses by V2C3-20-SKT05 and V2C3-30-SKT05 bispecifics along with parent SKT05 antibody and V2C3 nanobody. Full neutralization data provided in supplemental fig. S7. (B) Dendrogram of neutralization. Geometric mean PRTN_50_ are provided after the name of each antibody/nanobody/bispecific. Scale bar for 10 amino acid substitution per 100 sites is shown.

We further tested the top two bispecific nanobody-antibody constructs, V2C3-20-SKT05H, and V2C3-30-SKT05H, for their ability to neutralize diverse alphaviruses. With arthritogenic alphaviruses, ONNV, RRV, UNAV, MAYV, Sindbis virus (SINV), Semliki Forest virus (SFV), and CHIKV-LR, the parent SKT05 antibody was unable to neutralize any except for SINV. The parent V2C3 monovalent nanobody^36^ was able to neutralize ONNV, RRV, UNAV, MAYV, and SFV, achieved only weak neutralization of CHIKV-LR, and was unable to neutralize SINV. By contrast, both bispecifics potently neutralized all tested arthritogenic alphaviruses with an IC_50_ value of 0.006 μg/ml or less, except for SINV, against which it showed moderate neutralization (Figure 3A, Figure S7).

With encephalitic alphaviruses, we tested neutralization against live, authentic EEEV, WEEV, and two strains of VEEV, subtypes IAB and IIIA, the latter of which is also known as Mucambo virus. The parent SKT05 and V2C3 were generally unable to neutralize the viruses, but the bispecifics, especially V2C3-30-SKT05H, showed neutralization, against even EEEV and VEEV IIIA, which were not neutralized by either parent molecule (Figure 3A, Figure S7). Interestingly, little difference was found in neutralization potency between 20- and 30-linker versions of the bispecifics except for VEEV IIIA, for which only the 30-linker showed moderate neutralization; these results suggest that the impact of linker length on potency may be most apparent when both parent reagents have neutralization that is too weak to measure, when subtleties of on- and off-rates may be most impactful.

Correlations between binding and pseudovirus neutralization were low, except for the combined WEVEEV assessment (Fig. S8A). The modest correlations observed between ELISA binding and pseudovirus neutralization for individual encephalitic alphaviruses (WEEV: *r* = 0.42, *p* = 0.028; EEEV: *r* = 0.32, *p* = 0.104; VEEV: *r* = −0.29, *p* = 0.147; Figure S8A) reflect the limited dynamic range of neutralization IC_50_ values within each virus group. However, when all three encephalitic viruses were combined, spanning a 100-fold range in neutralization potency, a strong positive correlation emerged (*r* = 0.59, *p* < 0.0001). This pattern indicates that binding affinity and pseudovirus neutralization correlate over sufficiently large ranges of both parameters, but do not strongly predict each other within narrower subsets of constructs. Notably, we observed generally low correlations between authentic virus neutralization and either ELISA binding or pseudovirus neutralization for the best bispecific constructs, SKT05, and V2C3 (Fig. S8B–D), suggesting that additional viral or host factors beyond binding affinity constrain authentic virus neutralization. Such discrepancies are not uncommon, as pseudotyped platforms may not fully recapitulate the complex quaternary structures or entry kinetics of authentic virions. Consequently, instead of filtering candidates by mathematical correlation across the entire dataset, we selected our top two lead candidates based on a stringent dual-validation approach, prioritizing molecules that independently achieved top-tier potency thresholds in both the pseudovirus and live virus assays. Overall, the V2C3-30-SKT05 bispecific showed more potent neutralization than the 20-linker version, with near pan-neutralization of all 11 tested human-infecting alphaviruses (Figure 3B).

### Trispecific assessment

Considering the enhanced neutralization potency observed with V2C3-30-SKT05H versus the parent nanobody and antibody (Figure 3), we asked whether the addition of a second nanobody would enhance neutralization further. The V3A8f nanobody binds in a manner that is compatible with both V2C3 nanobody and SKT05 antibody; further, addition of V3A8f to the C-terminus of the SKT05 light chain (SKT05L-25-V3A8f) yielded the most potent pseudovirus neutralization of EEEV observed among all of the 23 bispecifics tested (Figure 2A). Co-transfection with V2C3-30-SKT05H and SKT05L-25-V3A8f followed by protein A purification yielded a trispecific, which we named SKT05-V2C3-V3A8f, with V2C3 attached to the N terminus of the SKT05 heavy chain and V3A8f to the C terminus of the light chain (Fig. S9A).

Pseudovirus neutralization by SKT05-V2C3-V3A8f trispecific was similar to that of the V2C3-30-SKT05H bispecific for VEEV, and about 2-fold less potent for WEEV and VEEV IIIA (Fig. S9B). Neutralization of chimeric viruses that express the non-structural proteins of SINV and the structural proteins of either VEEV or EEEV showed a small increase in potency for the trispecific (~2-fold) (Fig. S9C). Overall, the trispecific neutralized similarly to the bispecific. Thus, although a substantial increase in potency was achieved by adding a nanobody to SKT05, addition of a second nanobody to the bispecific did not achieve the same effect.

### Eleven tested alphaviruses provide near pan-coverage of alphaviruses that infect humans

**Figure 4.**
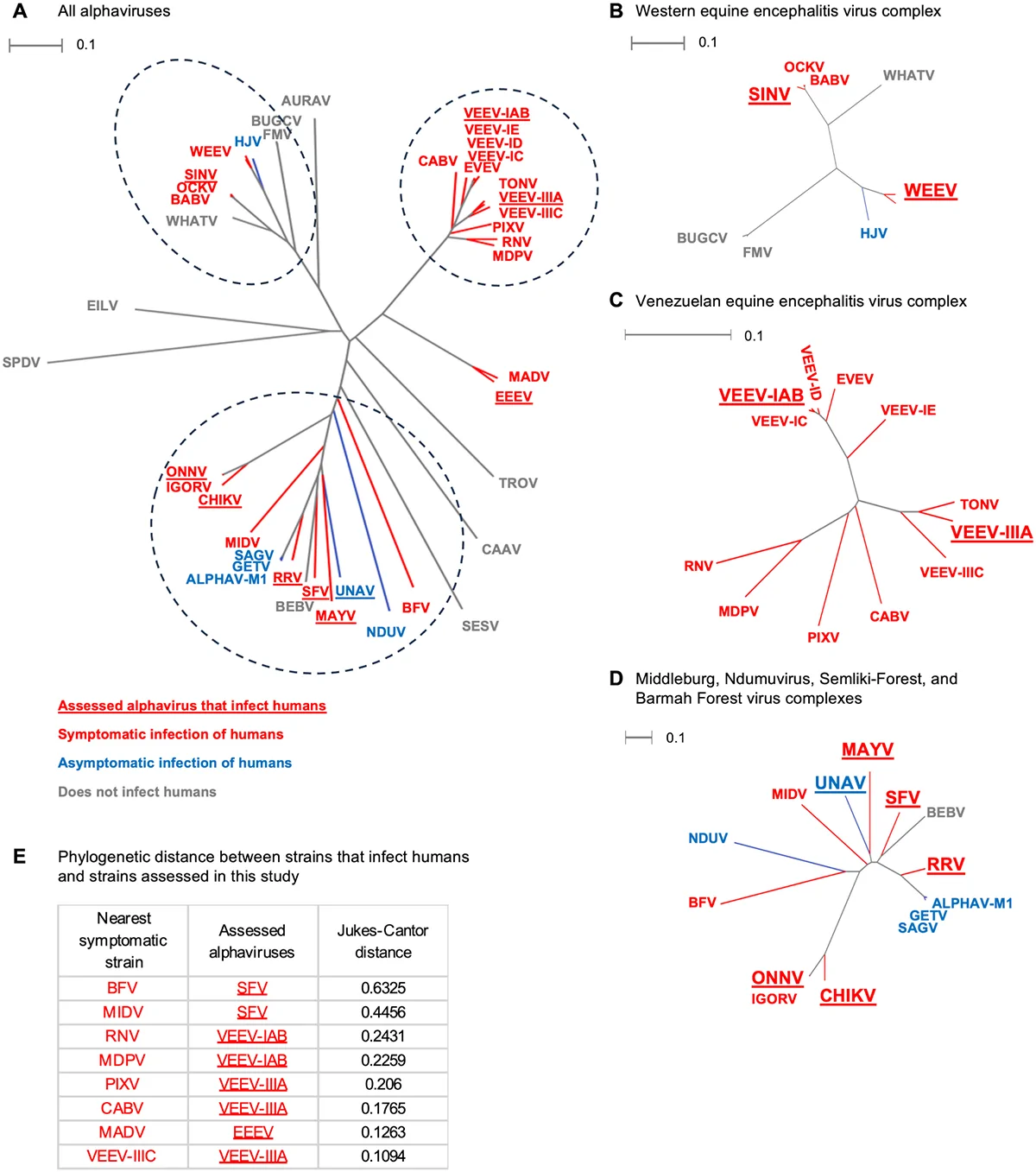
Eleven alphaviruses tested provide near-pan neutralization coverage of alphaviruses that infect humans. (A) Alphavirus phylogenetic tree. The viruses causing symptomatic and asymptomatic disease in humans are colored in red and blue, respectively. Eleven that are underlined are tested in this study. (B-D) Phylogenetic sub-trees of alphavirus complexes circled on the panel a (colored similarly). (E) Jukes-Cantor distances between the strains assessed in this study and the nearest symptomatic strains that infect humans. Phylogenetic analysis is done using E1 and E2 genes. Additional information on these alphaviruses is provided in table S1.

To assess the coverage of 11 tested alphaviruses with respect to all alphaviruses that infect humans, we compiled reported cases of alphavirus that do or do not infect humans, as well as those that induce symptoms. Of the 32 alphaviruses described, 26 could infect and induce symptoms in humans (Table S1). We then assessed the phylogenetic distance between all alphaviruses that induce symptoms in humans, and the 11 tested alphaviruses. Notably, 24 of 26 that infect and induce symptoms in humans were within a Jukes-Cantor distance calculated using E1 and E2 genes of less than 0.25 from one of the 11 alphaviruses we tested for neutralization. We acknowledge that genetic distance alone cannot fully predict complex antigenic relationships or guarantee cross-neutralization. However, this distance falls in the reliable range (0.05–0.3) for the Jukes-Cantor distance estimates. Only Barmah Forest virus (BFV) and Middelburg virus (MIDV) were phylogenetically more distal with Jukes-Cantor distances of 0.63 and 0.45, respectively (Figure 4). BFV and MIDV represent phylogenetically distinct clades within the alphavirus genus with lower documented disease burden in humans and thus may not represent substantial therapeutic gaps.

### *In vivo* efficacy of V2C3-30-SKT05 bispecific versus SKT05 with CHIKV

**Figure 5.**
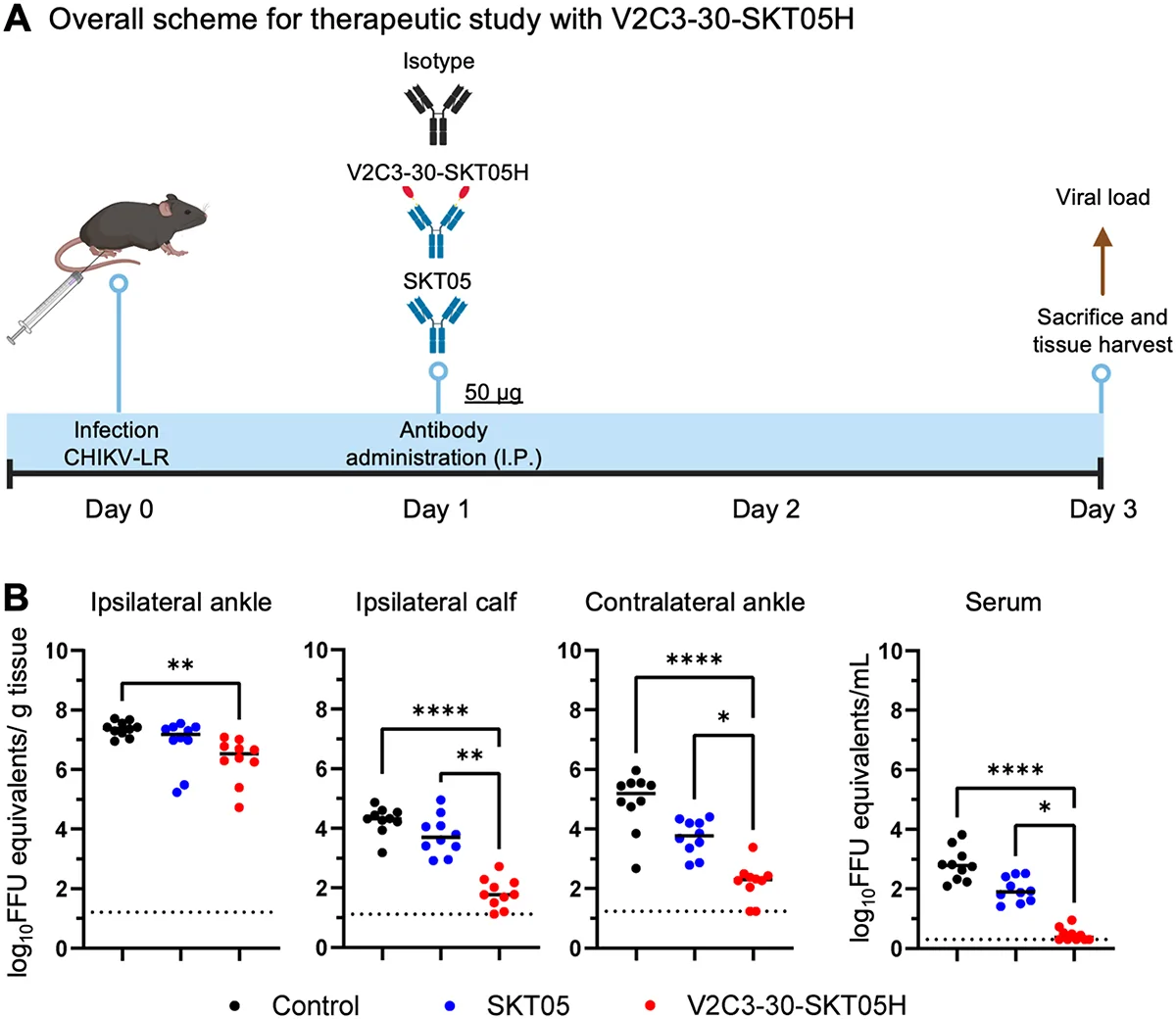
V2C3-30-SKT05H provides therapeutic protection *in* *vivo* against CHIKV. (A) Experimental schematic of antibody or bispecific therapeutic administration during CHIKV infection. C57BL6/J mice were subcutaneously infected with 10^3^ focus forming units (FFUs) of CHIKV-LR on Day 0. At day 1 post-infection, 50 µg of SKT05, V2C3-30-SKT05H, or an isotype control antibody was administered by intraperitoneal injection. At 3 days post-infection, animals were humanely euthanized, perfused, and tissues were harvested to determine viral RNA load. (B) viral load was determined by RT-qPCR in the ipsilateral ankle, ipsilateral calf, contralateral ankle, and serum (*n* = 10 mice/group; two independent experiments; Kruskal-Wallis with a Dunn’s posttest comparing all groups). The median is represented, and the dotted line indicates the limit of detection of the assay.

With enhanced neutralization capacity of V2C3-30-SKT05H against encephalitic and arthritogenic alphaviruses, we next assessed the therapeutic efficacy of the antibody-nanobody bispecific against a representative arthritogenic alphavirus, CHIKV. We challenged 4-week-old C57BL/6J mice with 10^3^ focus forming units (FFU) of CHIKV [strain La Reunion (LR)] subcutaneously in the rear footpad (Figure 5A). Previous studies showed SKT05-mediated protection against CHIKV infection when 200 µg was administered one day before infection.^28,39^ To better assess the efficacy of V2C3-30-SKT05H compared to SKT05, we used a dose-sparing approach during therapeutic delivery. At one day post-infection, we administered 50 µg of V2C3-30-SKT05H, SKT05, or an isotype control by intraperitoneal injection. At three days post-challenge, mice were euthanized, and tissues were harvested to assess viral load by RT-qPCR. Administration of V2C3-30-SKT05H significantly reduced viral burden in the ipsilateral ankle, contralateral ankle, ipsilateral calf, and serum compared to the isotype control treatment (Figure 5B). In contrast, mice receiving SKT05 had tissue viral load in the ipsilateral ankle and ipsilateral calf more similar to isotype control-treated animals with a trend toward reduced viral burden in the contralateral ankle and serum, although not statistically significant (Figure 5B). While there was no statistically significant difference between SKT05 and V2C3-30-SKT05H in the ipsilateral ankle, which is at the site of initial infection, V2C3-30-SKT05H showed statistically significant improvement over SKT05 in the ipsilateral calf, contralateral ankle, and serum.

To determine if V2C3-30-SKT05H extended the therapeutic window against CHIKV, we tested administration of 200 µg at two days post-infection and collected tissues at three and five days post-infection to assess viral load by RT-qPCR. The antibody dose was increased to account for increased CHIKV load in the tissues. V2C3-30-SKT05H provided significant reduction in CHIKV RNA compared to the isotype control, though significance versus SKT05 itself was only observed for the ipsilateral calf at three days post-infection (Fig. S10). Overall, V2C3-30-SKT05H provided enhanced therapeutic protection against CHIKV challenge.

### V2C3-30-SKT05H bispecific disrupts VEEV-VLP organization

**Figure 6.**
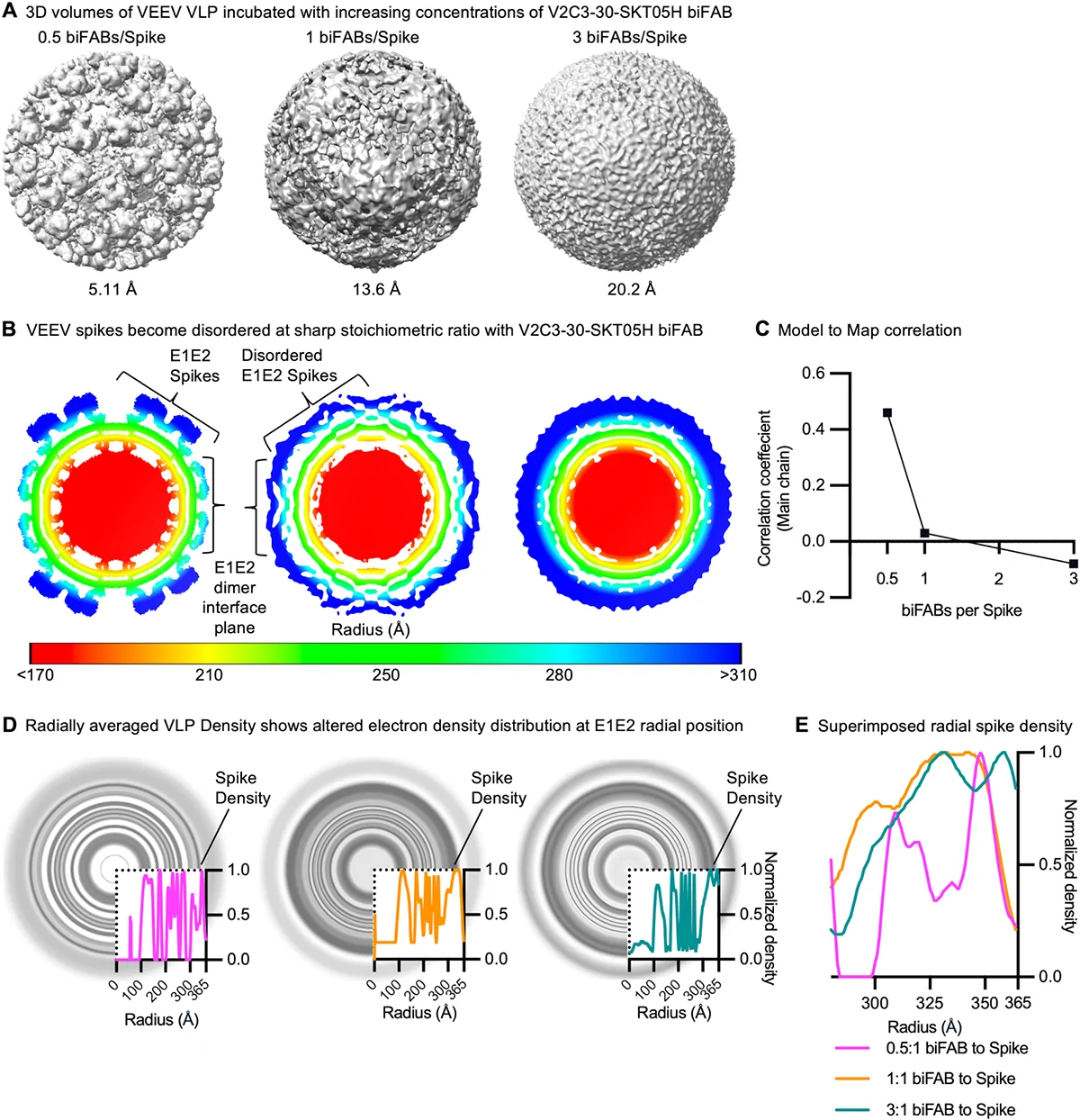
V2C3-30-SKT05H disorders E1E2 at a sharp stoichiometric boundary. (A) Cryo-EM reconstructions of VEEV VLP after incubation with V2C3-30-SKT05H Fab at a stoichiometric ratio of 0.5, 1, and 3 biFabs per Spike demonstrates a sharp boundary for VLP becoming disordered. (B) Density map slices of VEEV VLPs after incubation with V2C3-30-SKT05H biFAB showing radial position of VLP features. E1E2 spikes are visible in the lowest V2C3-30-SKT05H biFAB condition but become disordered when exposed to higher concentrations. (C) Average main chain correlation coefficient for asymmetric unit of E1E2 and capsid protein fit into map of VEEV treated with 0.5 molar ratio of V2C3-30-SKT05H biFAB and aligned to the same symmetry axis of maps from VEEV treated with 1, and 3 molar ratio of V2C3-30-SKT05H biFAB. (D) Gray scale images of radially averaged cryo-EM maps for VEEV VLPs treated with different concentrations of V2C3-30-SKT05H biFAB show altered electrostatic potential density distribution at the E1E2 spike radial position. (E) Superposition of normalized radial density calculated in (d) for the three conditions illustrates differential density distribution at the radial position of E1E2 spike. (for details on cryo-EM maps – see fig. S10 and table S2.) although different number of particles were used in map calculations, the quality of the resultant maps was similar allowing comparison.

To gain insight into the impact of V2C3-30-SKT05H binding, we analyzed cryogenic electron microscopy (cryo-EM) data (Figure 6 and Figure S11) on the bispecific binding to VEEV VLPs, using the antigen-binding fragment (Fab) of the V2C3-30-SKT05H bispecific, which comprised the Fab of SKT05 with a V2C3 attached to the heavy chain N terminus, referred to here as a “biFAB.” VEEV VLP was incubated with V2C3-30-SKT05H biFAB at molar ratios of 0.5, 1, and 3 biFABs per E1E2 trimeric spike. At a ratio of 0.5 biFAB to 1.0 VEEV E1E2 spike, we observed a 5.11 Å structure of VEEV VLP, but did not observe any bound biFAB. At a ratio of 1.0 and 3.0, we observed highly disordered VEEV VLPs, with no discernable structure of biFAB. (Figure 6A and Figure S11). The three cryo-EM maps were then analyzed to view radial VLP features (Figure 6B). While the 0.5 ratio data set has radial features corresponding to E1E2 spike (blue/light blue) and capsid protein (green), only features of the capsid protein could be discerned for the higher ratio data sets. The corresponding radial position for these data sets showed only disordered density. The structure of the VEEV VLP asymmetric unit (capsid, E1E2, PDB 8DEC) was then fit into the cryo-EM map of the 0.5 ratio data set, aligned to the same symmetry axis as the higher ratio data sets, and model to map correlation coefficients were calculated for the main chain (Figure 6C). While the 0.5 ratio had a high correlation coefficient, the correlation coefficients for the 1.0 and 3.0 ratio data sets were near 0 or negative, indicating a lack of structural features. The three maps were then radially averaged, and the radially averaged density was displayed as a gray scale image and normalized radial density was plotted for each data set (Figure 6D). Analysis of the radial position of the E1E2 spike trimer (Figure 6E) demonstrated an altered radial distribution of electrostatic potential, Overall, these data indicate a sharp stoichiometric boundary (between 0.5 and 1 molar) where V2C3-30-SKT05H induces disorder of the E1E2 spikes, disorder suggesting an explanation for the potent neutralization by this designed bispecific.

## Discussion

In this study, we linked a nanobody (V2C3) to the heavy chain of an antibody (SKT05) to create an antibody-nanobody bispecific (V2C3-30-SKT05H) capable of near pan-neutralization of alphaviruses that infect humans. We used lentiviral pseudotyped viruses expressing alphavirus envelope proteins for our initial screening of 23 bispecifics, as this system allows rapid, high-throughput assessment in a biosafety level (BSL)-2 setting. However, we recognized that pseudovirus neutralization does not always correlate strongly with live-virus neutralization, as we observed here (Fig. S8). Alternative approaches, including SINV-based chimeric viruses,^40^ chimeric VSV/WEEV pseudoviruses,^41^ and TC83 (live-attenuated VEEV) for plaque reduction neutralization testing,^42^ have been reported to show comparable or higher correlation with authentic virus neutralization in some contexts. Despite the modest pseudovirus-to-live-virus correlation observed in our study, the lentiviral pseudovirus platform was valuable for the initial down-selection of candidates from 23 constructs to 10, after which we prioritized live-virus testing to identify the most potent bispecifics. This tiered screening approach balanced practical constraints (throughput, BSL containment, cost) with the need for authentic virus validation, which we prioritized for lead candidates. The extensive and potent live virus neutralization was unexpected, based on the neutralization of the parent antibodies, especially amongst the encephalitic alphaviruses, VEEV-IIIA and EEEV, which were not neutralized by either SKT05 or V2C3. We note in this context that SKT05 does not neutralize live virus, though it does protect from VEEV and CHIKV challenge,^28^ suggesting that the protection provided by the antibody-nanobody bispecific to be enhanced. We confirmed this in therapeutic murine studies with CHIKV (Figure 5B), where the bispecific, but not the parent SKT05 antibody, showed significant reduction in viral titers versus an isotype control. While the results of the animal experiments cannot be sex-stratified due to insufficient numbers of each sex to power statistical analysis, similar results were observed between male and female mice. SKT05 protects against alphavirus challenge through Fc-dependent mechanisms in the absence of neutralizing activity.^39^ The V2C3-30-SKT05H bispecific retains SKT05‘s Fc functionality while adding direct neutralizing capacity via V2C3-30-SKT05H-mediated E1E2 spike disassembly (Figure 6). This combination of Fc-dependent immune activation and direct neutralization likely explains the superior *in* *vivo* efficacy of V2C3-30-SKT05H compared to SKT05 alone (Figure 5B), suggesting that nanobody introduction enhances the therapeutic profile by providing dual mechanisms of viral inhibition.

The difference in neutralization between pseudovirus neutralization and live-virus neutralization was also pronounced, and in general we observed little correlation between the two, except that the viruses, which neutralized in the live virus context, generally also neutralized in the pseudovirus context (but not vice-versa). In pseudovirus neutralization, all three nanobodies showed similar activities; however, in live-virus neutralization, no neutralization was observed for bispecifics with nanobodies V2B3 or V3A8f, only for nanobody V2C3, which showed potent live-virus neutralization in two different formats, attached to either the N terminus of the heavy chain or to the C terminus of the light chain of SKT05. These subtleties in function suggest that it may be helpful to screen experimentally bispecific panels of the best individual antibodies and nanobodies to identify potent and broad neutralizers, as we have done here with SKT05 and three nanobodies. Perhaps relevant to this, the potent neutralization we observed with V2C3-30-SKT05H appears to arise from the ability of this bispecific to induce trimer disorder at low ratios of antibody to spike (Figure 6), a disorder not observed with Fabs of only SKT05,^28^ because of the dual epitope-binding capacity of the bispecific.

In addition to SKT05 and the three nanobodies, V2B3, V2C3, and V3A8f, explored in this study, other broadly reactive antibodies against alphaviruses have been identified and their structural recognition defined. These include a collection of broadly neutralizing antibodies against alphaviruses,^30^ CHK-265 protected against CHIKV (PDB 6W09), RRV (PDB 6VYV), and MAYV (PDB 6W1C, 6W2U),^43^ and broadly reactive a-EEV monoclonal antibodies DC2.112, DC2.315, EEEV-138, EEEV-179, and EEEV-346,^34,44^ as well as tri-specific antibodies SKT01, SKT02, SKT04, SKT14, SKT15, SKT19, SKT20 (PDB 8EEV), and SKT23 protected against WEEV, EEEV, and VEEV.^28^ The bispecific approach outlined in this study might provide utility in combining these other broadly reactive antibodies and nanobodies. In this context, we note that bispecific antibodies in general may face manufacturing hurdles from having two heavy and two light chains, with multiple potential pairings, although multiple technologies have been developed^45^ that enable bispecific IgG engineering and manufacturing to solve this cognate pairing challenge. Antibody-nanobody bispecifics, however, made by appending the nanobody to one of the antibody chains, retains the single heavy chain and single light chain of the parent antibody – just that one of these chains are modified to add a nanobody – easing manufacturing. For example, we recently manufactured the CAP256.J3LS antibody-nanobody and found the bispecific expresses at a yield of ~2 g/L, more than the parent antibody by itself. This antibody-nanobody showed a half-life in human FcRn knock-in mice that was better than either of the parents.^37^

The phylogenetic breadth achieved by V2C3-30-SKT05H – neutralizing 11 of 11 tested alphaviruses spanning four distinct antigenic complexes – demonstrates that pan-reactive therapeutic antibodies are achievable against this challenging viral family. This breadth encompasses the viruses responsible for the majority of alphavirus disease burden globally, including CHIKV, RRV, MAYV, and VEEV. The bispecific’s *in* *vivo* efficacy in a CHIKV mouse model validates that *in* *vitro* neutralization breadth translates to protective capacity. These findings suggest that rational combination of complementary epitopes can overcome the antigenic complexity that has historically limited the breadth of single-antibody therapeutics against alphaviruses.

## Materials and methods

### Structure-based design of antibody-nanobody bispecifics

The design of SKT05-nanobody bispecifics was based on the structures of SKT05-VEEV (PDB ID 8EEU),^28^ V2B3-VEEV (PDB ID 9NRX), V2C3-VEEV (PDB ID 9NRY), and V3A8f-VEEV (PDB ID 9NRZ). To design the V2B3-SKT05 bispecific, we completed the structures of SKT05-VEEV and V2B3-VEEV spikes to fill the asymmetric unit of the *T* = 4 icosahedral particle, corresponding to the complete structure of VEEV VLP. Next, the icosahedral symmetry operations of Chimera software^46^ were applied to generate 240 copies of symmetry-related VEEV VLP, SKT05, and V2B3 molecules. Six spikes grouped around the icosahedral 2-fold axis enabled the evaluation of all possible arrangements of SKT05 and V2B3 bound near each other. Two shortest distances between the N- and C-termini of the V2B3 nanobody and the N and C termini of the SKT05 antibody were identified to estimate the length of the bispecifics (ggsgg)_n_ linker. Based on the two shortest distances, a linker range was calculated using (ggsgg)_n_ peptides, where the smallest n was chosen so that n * 3.8 Å * 5 would be greater than the shortest distance, and the largest n was chosen so that n * 2.0 Å *5 would be greater than the second shortest distance. The design of the V2C3-SKT05 and V3A8f-SKT05 bispecifics was done similarly. The figures were generated using Chimera^46^ and Pymol (Schrodinger Inc).

### Antibody expression and purification

Bispecific antibody-nanobody and regular SKT05 antibody genes were synthesized (Gene Universal Inc.). Antibody-expressing genes were cloned into pVRC8400 expression vector. For antibody production, 150 μL of Turbo293 transfection reagent (Speed BioSystems) were mixed with 2.5 mL Opti-MEM medium (Thermo Fisher) and incubated for 5 min at room temperature (RT). 50 μg of antibody expressing plasmid DNAs (25 μg of SKT05 heavy chain and 25 μg of SKT05 light chain-nanobody bispecific, or 25 μg of SKT05 light chain and 25 μg of SKT05 heavy chain-nanobody bispecific) were added to 2.5 mL of Opti-MEM medium in another tube. The diluted transfection reagent was diluted into 2.5 mL Opti-MEM medium containing plasmid DNAs. Transfection reagent and DNA mixture was incubated for 15 min at RT, and added into 40 mL of Expi293 cells (Thermo Fisher) at 2.5 million cells/mL. The transfected cells were incubated in a shaker at 120 rpm, 37°C, 9% CO_2_. After 5 days of incubation, antibodies in centrifuge-clarified supernatants were purified over 0.5 mL Protein A (Cytiva) resin in columns. Antibody was eluted from Protein A columns with IgG elution buffer (Pierce), immediately neutralized with one-tenth volume of 1 M Tris-HCl pH 8.0. The antibodies were dialyzed in phosphate-buffered saline (PBS), adjusted concentration to 0.5 mg/mL and filtered (0.22 μm) for neutralization assays. Antibody Fabs are purified through size exclusion chromatography columns. 0.5 mg (1 mg/mL) antibody Fab was injected onto the Superose 6 Increase column (Cytiva) on a BioRad (Hercules, CA) NGC Chromatography System Quest 10. The flow rate was set to 0.5 mL/min and the mobile phase was 1 ×PBS (Thermo Fisher).

#### Production and neutralization of WEEV, EEEV, and VEEV env-pseudotyped lentiviral reporters

We produced EEV Env-pseudotyped lentiviral reporters that express a luciferase reporter gene to assess neutralization of WEEV, EEEV, and VEEV in a BSL-2 setting as previously described.^19,28^ Briefly, 293T cells were transfected with 500 ng of WEEV, EEEV, or VEEV envelope plasmid encoding the E2 and E1 glycoproteins (CBA87, PE-6, and TC-83 strain, respectively), 7 µg of a transducing vector encoding a luciferase gene under the control of a cytomegalovirus (CMV) promoter (pHR’ CMV-luciferase plasmid), and 7 µg of a packaging plasmid that expresses all HIV-1 structural proteins except envelope (pCMVDR8.2). Recombinant lentiviral vectors were transfected using Fugene 6 (Promega) and culture medium was completely changed after overnight incubation. Supernatants were harvested 48 hours after culture medium change, followed by filtering through a 0.45 mm syringe filter. Aliquots were stored frozen at −80°C.

Neutralization assays were performed as described previously.^28^ Briefly, one day before infection tissue culture treated black and white 96-well isoplates (PerkinElmer) were seeded with 10^4^ cells/well of 293A cells in Minimum Essential Media (MEM, ThermoFisher) supplemented with 5% fetal bovine serum (FBS) and 1% penicillin-streptomycin (M5 media). Titrated amounts of Env-pseudotyped lentiviral reporters (p24 levels ~50 ng/mL) were incubated with 4-fold serially diluted monoclonal antibody for 25 minutes at 37°C in a 5% CO_2_ incubator. The anti-SIV antibody ITS103.01 served as a negative control.^47^ Next, virus:monoclonal antibody solution was added to 293A cells and incubated for 2 hours at 37°C with 5% CO_2_. After incubation, M5 media was added to the plate and allowed to incubate overnight. Approximately 24 hours after M5 media addition cells were lysed using cell lysis buffer (Promega). Luciferase activity was measured using the Micro-Beta JET (PerkinElmer) after incubation with a luciferase assay reagent (Promega) according to the manufacturer’s protocol. All antibodies were performed in triplicate for an individual experiment. Inhibition values were calculated as follows: inhibition (%) = [1 – (luciferase activity (cps) in pseudotyped lentiviral reporter–infected cells incubated with the indicated dilutions of antibody) / (luciferase activity (cps) in pseudotyped lentiviral reporter–infected cells incubated with the same dilutions of control antibody)] X 100. If the dilutions were not sufficient to define the neutralization curve, the neutralization was repeated with additional dilutions. Calculations for the 50, 80, and 90% inhibitory concentrations were made with GraphPad Prism (Graphpad Software).

### Live virus

All PRNTs except ONNV: Antibody was diluted at an initial concentration of 100 µg/mL in minimum essential media eagle (MEM) with 2% heat inactivated-fetal bovine serum (HI-FBS), 1% HEPES, and 2% Pen/Strep and serial 1:2 dilutions were made. Virus stocks were diluted to a concentration of 2.0 × 10^3^ PFU/mL and added 1:1 to the serially diluted samples (resulting in a 50 µg/mL initial dilution) or control wells containing media alone for the virus-only control. Samples were incubated overnight at 4°C. Six-well plates of Vero 76 cells were infected with 0.1 mL of each serial dilution per well in duplicate and plates were incubated at 37°C for 1 hr. After 1 hr incubation, cells were overlaid with 0.6% agarose in Basal Medium Eagle (BME) with 10% HI-FBS, and 2% Pen/Strep, and incubated for ~24 hrs at 37°C, 5% CO_2_. A second overlay containing 0.6% agarose in BME with 10% HI-FBS, 2% Pen/Strep, and 4% of total volume neutral red vital stain was added to the wells and further incubated for 18–28 hours to visualize plaques. Plaques were counted following incubation with stain overlay. The virus-only control was counted, and the endpoint titer was determined to be the highest dilution ≥ 50% reduction (PRNT_50_) or ≥80% reduction (PRNT_80_) in the average number of plaques observed from the duplicates relative to virus-only control wells. The limit of detection for the antibodies was 50 µg/mL. Virus-specific ATCC hyperimmune mouse ascites fluid was utilized as a positive control (VEEV Cat#VR-1249AF; EEEV Cat#VR-1242AF; WEEV Cat#VR-1251AF; CHIKV Cat#VR-1241AF; RRV Cat#VR-1246AF; SFV Cat#VR-1247AF; MAYV Cat#VR-1277AF; SINV Cat#VR-1248AF), except for UNAV as an UNAV-specific hyperimmune mouse ascites fluid was not commercially available. For UNAV, a CHIKV-specific ATCC hyperimmune mouse ascites fluid was utilized. sdAb ACVE^48^ was used as a negative control.^48^ The ascites fluid comes from ATCC’s legacy NIH research stocks, which were generated during 1970s-1980s as part of the arbovirus reference programs.

ONNV PRNT: Antibody was diluted at an initial concentration of 100 µg/mL in MEM with 2% HI-FBS, 2% HEPES, 2% L-glutamine/GlutaMAX, and 2% Pen/Strep and serial 1:2 dilutions were made. Virus stocks were diluted to a concentration of 2.0 × 10^3^ PFU/mL and added 1:1 to the serially diluted samples (resulting in a 50 µg/mL initial dilution) or control wells containing media alone for the virus-only control. Samples were incubated overnight at 4°C. Six-well plates of Vero CCL-81 cells were infected with 0.1 mL of each serial dilution per well in duplicate and plates were incubated at 37°C for 1 hr. After 1 hr incubation, cells were overlaid with 0.6% agarose in BME with 20% HI-FBS, 2% non-essential amino acids, 2% L-glutamine/GlutaMAX, and 2% Pen/Strep, and incubated for ~48 hrs at 37°C, 5% CO_2_. A second overlay containing 0.6% agarose in BME with 10% HI-FBS, 2% non-essential amino acids, 2% L-glutamine/GlutaMAX, 2% Pen/Strep, and 4% of total volume neutral red vital stain was added to wells and the plates were further incubated for 18–28 hrs for visualization of plaques. Plaques were counted following incubation with stain overlay. Plaques were counted following incubation with stain overlay. The virus-only control was counted, and the endpoint titer was determined to be the highest dilution ≥ 50% reduction (PRNT_50_) or ≥80% reduction (PRNT_80_) in the average number of plaques observed from the duplicates relative to virus-only control wells. The limit of detection for the antibodies was 50 µg/mL. A CHIKV-specific ATCC hyperimmune mouse ascites fluid was utilized for a positive control as an ONNV-specific hyperimmune mouse ascites fluid was not commercially available. sdAb ACVE^48^ was used as a negative control.

### *In vivo* assessment of bispecific efficacy against CHIKV

Experiments were performed in accordance with the recommendations in the Guide for the Care and Use of Laboratory Animals of the National Institutes of Health in compliance with the National Institute of Allergy and Infectious Diseases Animal Care and Use Committee under the approved protocol LVD-6E. All studies with CHIKV were performed under BSL3/ABSL3 conditions. CHIKV (strain La Reunion_OPY1) was generated from a cDNA clone, and passaged three times on C6/36 cells, as previously described.^49^ Four-week-old wild-type C57BL/6J male and female mice were purchased from Jackson Laboratory. Mice were maintained on a 14 h light/ 10 h dark cycle in individually ventilated cages under barrier and specific-pathogen-free conditions with no more than 5 mice per cage. Mice received nestlets and wooden chew sticks for enrichment. The temperature and humidity were maintained at 20–24°C and 30–70%, respectively. Mice were randomly placed in cages upon arrival. Equal numbers of male and female mice were used in the experimental groups. Mice were inoculated in the rear footpad with 10^3^ FFU of CHIKV in Hanks Balanced Salt Solution (HBSS) supplemented with 1% heat-inactivated FBS under isoflurane anesthesia. SKT05-LS, V2C3-30-SKT05H, or an isotype control (ITS103.01), prepared in PBS at a dose of 50 µg was administered by intraperitoneal injection at 1 dpi or 200 µg was administered by intraperitoneal injection at 2 dpi. At 3 or 5 dpi, mice were euthanized by overdose of the anesthetic 2, 2, 2-Tribromoethanol and exsanguination, perfused with PBS, and indicated tissues were collected to determine viral RNA load. Thirty mice (10 mice/group) were used in the 50 µg dose study and 74 mice were used in the 200 µg dose studies (8 mice/group were harvested on 3 dpi and 10 mice/group were harvested on 5 dpi) across 2 independent experiments, for a total of 84 mice used in this study. Sample size for each time point was determined based on prior experiments to ensure the analysis was appropriately powered for statistical analysis.

Perfused tissues were homogenized in 1 mL of viral infection medium [DMEM supplemented with 2% heat-inactivated FBS (HI-FBS; Omega Scientific, Inc.), 10 mM HEPES (Gibco), and 100 U/mL of penicillin and streptomycin (Gibco)] using 1 mm diameter Zirconia/Silica beads (BioSpec products) in a MagNA Lyser (Roche) for 60s at 6,000 rpm. For viral RNA load analysis, homogenates were clarified by centrifugation at 10,000 rpm for 5 min. RNA was extracted from the clarified homogenate using the Kingfisher Duo Prime with MagMax-96 Viral RNA isolation kit (Thermo Fisher) following the manufacturer’s instructions. To determine viral burden, equal quantities of RNA were added to TaqMan fast virus 1-step master mix (Thermo Fisher) with CHIKV-LR E1 specific primers/probes (Forward: 5’- TCGACGCGCCCTCTTTAA −3,’ Reverse: 5’- ATCGAATGCACCGCACACT-3,’ Probe:5’-6FAM/ACCAGCCTG/ZEN/CACCCATTCCTCAGAC/3IABkFQ −3’). Reactions were run on a QuantStudio 3-Real-Time PCR System (Applied Biosystems) and viral RNA isolated from CHIKV viral stocks were used to generate a standard curve based on FFU equivalents. All tissues were normalized to gram of tissue or mL of serum. Statistical analysis was performed using GraphPad Prism (Version 10.6.1) and significance was determined using a Kruskal-Wallis test with a Dunn’s posttest comparing all groups (*p < 0.05, **p < 0.01, ***p < 0.001, ****p < 0.0001).

### Focus reduction neutralization test

The SINV-VEEV Trinidad Donkey and SINV-EEEV FL93-939 chimeric viruses were generously provided by Dr. William Klimstra (University of Pittsburgh) and passaged once on Vero cells. Focus reduction neutralization tests were performed as previously described,^28^ starting at a concentration of 50 µg/ml with 4-fold serial dilutions. Wells containing the antibodies were compared to wells with no antibody to determine the percent relative infection. The IC_50_ value was determined using nonlinear regression with the top and bottom constrained to 100 and 0, respectively.

### Cryo-EM analyses of bispecific-VEEV VLP complexes

Cryo-EM Grid Preparation. Samples for cryo-EM grid preparation were produced by first mixing 20 µl of purified VEEV VLP at 0.14 mg/mL with 1–2 µl of V2C3-30-SKT05H biFAB at 2.2 mg/ml, adjusted for molar ratios of 0.5, 1.0 and 3.0 biFABs per E1E2 spike. Cryo-EM grids were prepared by triple blotting. 3 μL of sample was applied to a freshly glow discharged carbon-coated copper grid (Quantifoil R2/2 + 2 nm C, Cu 300) and blotted twice by hand, with a 30 sec waiting period. The final blot and vitrification in liquid ethane were completed using a Vitrobot Mark IV with a wait time of 30 sec, a blot time of 3 sec, and a blot force of −8.

Image Processing. Cryo-EM data were collected on a Titan Krios operating at 300 keV, equipped with a K3 detector (Gatan) operating in counting mode. Data were acquired using Leginon.^50^ The dose was fractionated over 50 raw frames. For all structures, the movie frames were aligned and dose-weighted using cryoSPARC 4.6.2^51^; the CTF estimation, particle picking, 2D classifications, ab initio model generation, heterogeneous refinements, homogeneous 3D refinements and non-uniform refinement calculations were carried out using cryoSPARC 4.6.2.

Cryo-EM Map Analysis. Radial images of cryo-EM maps were generated using UCSF Chimera.^46^ To estimate correlation coefficients, PDB 8DEC was fit into cryo-EM maps using UCSF Chimera, and correlation coefficients were generated using Phenix.^52^

Radial Averaging. Radial averaging was adapted from a previously described method.^53^ Radial averaging was performed using the math.rotationalaverage function in EMAN2.^54^ Gray scale images were generating using UCSF Chimera, radial lines were traced using ImageJ,^55^ normalized using Microsoft Excel, and plotted using GraphPad Prism.

## Supplementary Material

SupMat_KMAB_2026_0143_R3_2026_08_26.docx

## Data Availability

The authors confirm that the data supporting the findings of this study are available within the article and its supplementary materials.
