# Supplementary material for: Near pan-alphavirus neutralization by an antibody-nanobody bispecific
Source: MAbs. 2026 Sep 23;18(1):2731281. doi: 10.1080/19420862.2026.2731281 (PMC13613895; doi:10.1080/19420862.2026.2731281)
Supplement: Supplementary file 1 [file KMAB_A_2731281_SM0275.docx]

Supplemental Figures and Tables

**Figure S1.** Design of V2B3-SKT05 bispecifics. (A) Structure of VEEV VLP (yellow) with modeled V2B3 nanobody (green) and SKT05 Fab (blue). Left, major icosahedral symmetry elements displayed on entire VLP. Right, the six spikes around the icosahedral 2-fold. (B) Zoom-in for SKT05 Fab, highlighting all distances less that 90 Å for either N terminus of antibody (heavy or light chain) to C terminus of nanobody or C terminus of antibody (light chain) to N terminus of nanobody. (C) Constructs and distances are shown for V2B3-SKT05 bispecifics. (ggsgg)_n_ linker peptide is gly-gly-ser-gly-gly, which can be repeated n times.

**Figure S2.** Design of V2C3-SKT05 bispecifics. (A) Structure of VEEV VLP (yellow) with modeled V2C3 nanobody (magenta) and SKT05 Fab (blue). Left, major icosahedral symmetry elements displayed on entire VLP. Right, the six spikes around the icosahedral 2-fold. (B) Zoom-in for SKT05 Fab, highlighting all distances less that 90 Å for either N terminus of antibody (heavy or light chain) to C terminus of nanobody or C terminus of antibody (light chain) to N terminus of nanobody. (C) Constructs and distances are shown for V2C3-SKT05 bispecifics. (ggsgg)_n_ linker peptide is gly-gly-ser-gly-gly, which can be repeated n times.

**Figure S3.** Design of V3A8f-SKT05 bispecifics. (A) Structure of VEEV VLP (yellow) with modeled V3A8f nanobody (cyan) and SKT05 Fab (blue). Left, major icosahedral symmetry elements displayed on entire VLP. Right, the six spikes around the icosahedral 2-fold. (B) (left) Zoom-in for SKT05 Fab, highlighting all distances less that 90 Å for either N terminus of antibody (heavy or light chain) to C terminus of nanobody or C terminus of antibody (light chain) to N terminus of nanobody. (right) Rotated zoom-in showing close contact, but no clash between V3A8f and SKT05. (C) Constructs and distances are shown for V3A8f-SKT05 bispecifics. (ggsgg)_n_ linker peptide is gly-gly-ser-gly-gly, which can be repeated n times.

**Figure S4.** Binding titers of designed SKT05-nanobody bispecifics. ELISA binding IC_50_ and IC_80_ titers against WEVEEV VLPs for the 23 SKT05-nanobody bispecific constructs and SKT05 IgG.

**Figure S5. Nanobody-SKT05 bispecific constructs bind VLP and neutralize pseudovirus.** (A) ELISA binding curves for V2B3-SKT05 (left), V2C3-SKT05 (middle), and V3A8f-SKT05 (right) constructs against WEEV (red), EEEV (blue), and VEEV (green) VLPs. (B) Pseudovirus neutralization curves for V2B3-SKT05 (left), V2C3-SKT05 (middle), and V3A8f-SKT05 (right) constructs against WEEV (red), EEEV (blue), and VEEV (green) pseudovirus. All data displayed as nonlinear fit of log-transformed values.

**Figure S6.** Arthritogenic live virus neutralization curves on designated SKT05-nanobody bispecifics. Percent neutralization curves for the 4 bispecific nanobodies that demonstrated neutralization against ONNV, RRV, UNAV, and MAYV. Assay was completed at least once and each dilution was done in duplicate.

**Figure S7.** Neutralization curves for the two lead bispecific antibodies against encephalitic and arthritogenic alphaviruses. Percent neutralization curves against 7 arthritogenic alphaviruses (ONNV, RRV, UNAV, MAYV, SINV, SFV, and CHIKV-LR) and 4 encephalitic alphaviruses (EEEV, WEEV, VEEV IAB, and VEEV IIIA). Assay was completed at least once and each dilution was done in duplicate.

**Figure S8.** Correlations between VLP-ELISA binding, pseudovirus neutralization, and real virus neutralization. (A) Comparisons between VLP-ELISA binding EC_50_ titers and pseudovirus neutralization IC_50_ titers for the 23 SKT05-nanobody bispecific constructs and SKT05 IgG against WEEV, EEEV, and VEEV. (B) Comparisons between VLP-ELISA binding EC_50_ titers and pseudovirus neutralization IC_50_ titers. (C) VLP-ELISA binding EC_50_ titers and authentic virus neutralization PRNT_50_ titers. (D) Pseudovirus neutralization IC_50_ titers and authentic virus neutralization PRNT_50_ titers for the top 2 SKT05-nanobody bispecific constructs, nanobody V2C3, and SKT05 IgG. Correlations were made by two-tailed Pearson test with a 95% confidence interval.

**Figure S9.** Construction and neutralization of a nanobody-SKT05 trispecific. (A) Trispecific schematic. (B) Neutralization of WEEV, EEEV, VEEV, and VEEV IIIA pseudoviruses. (C) Chimeric virus neutralization against SINV-VEEV and SINV-EEEV.

**Figure S10.** V2C3-30-SKT05H administration at 2 dpi reduces CHIKV burden in mice. (A) Experimental schematic of antibody or bispecific therapeutic administration during CHIKV infection. C57BL6/J mice were subcutaneously infected with 103 focus forming units (FFUs) of CHIKV-LR on Day 0. At day 2 post-infection, 200 μg of SKT05, V2C3-30-SKT05H, or an isotype control antibody was administered by intraperitoneal injection. (B) The ipsilateral foot was measured (height x width) with digital calipers at 5 dpi and normalized to measurements taken before infection (n = 10 mice/group; two independent experiments; one-way ANOVA with a Tukey’s post-test comparing all groups). The median is represented. (C) At day 3 and 5 post-infection, animals were humanely euthanized, perfused, and tissues were harvested to determine viral RNA load. Viral load was determined by RT-qPCR in the ipsilateral ankle, ipsilateral calf, contralateral ankle, and serum (3 days post-infection (dpi) only) (n = 8 mice/group for 3 dpi and n = 10 mice/group for 5 dpi; two independent experiments; Kruskal-Wallis with a Dunn’s post-test comparing all groups). The median is represented, and the dotted line indicates the limit of detection of the assay.

**Figure S11.** Cryo-EM validation of VEEV VLP. (A) Representative micrograph, representative 2D class averages, the gold-standard Fourier shell correlation for the VEEV VLP after global refinement, and heatmap showing the orientations of all VEEV VLP particles used in the global refinement for VEEV VLP treated with 0.5 molar ratio biFAB, (B) treated with 1 molar ratio biFAB, and c treated with 3 molar ratio biFAB.

**Table S1**. Alphavirus species and human disease.

This table provides classification of alphavirus species with additional epidemiological and clinical characteristics. The table includes geographic distribution, epidemic/endemic status, epizootic/enzootic classification, pathogenic potential (arthritogenic vs. encephalitic), and human disease associations. Eleven tested in this study are highlighted in bold red and underlined; those not tested but show symptomatic disease are in red; those that are asymptomatic are in blue; and those that are noninfectious for humans are in gray.

Column header details:

• Geographic Range: Primary regions where the virus is naturally found or has been isolated

• Epidemic/Endemic: Epidemic viruses cause sporadic outbreaks; endemic viruses maintain continuous circulation

• Epizootic/Enzootic: Epizootic viruses cause sporadic outbreaks in animals; enzootic viruses maintain continuous circulation in animal populations

• Arthritogenic/Encephalitic: Arthritogenic viruses primarily cause joint disease; encephalitic viruses primarily cause brain inflammation

• Human Disease: Yes indicates documented human infections; no indicates no known human disease; unknown indicates insufficient data

• Symptomatic/Asymptomatic: Symptomatic indicates clinically apparent disease; asymptomatic indicates subclinical infections; unknown indicates insufficient data

| **Virus abbreviation from Fig. 5** | **Species** | **Virus name** | **Geographic range** | **Epidemic/**  **Endemic** | **Epizootic/**  **Enzootic** | **Arthritogenic/**  **Encephalitic** | **Human disease** | **Symptomatic/**  **Asymptomatic** | **References** |
| --- | --- | --- | --- | --- | --- | --- | --- | --- | --- |
| AURAV | Alphavirus aura | Aura virus | South America | epidemic | epizootic | unknown | unknown | unknown | (1) |
| BFV | Alphavirus barmah | Barmah Forest virus | Australia, Papua New Guinea | endemic | enzootic | arthritogenic | yes | symptomatic | (2, 3) |
| BEBV | Alphavirus bebaru | bebaru virus | Malaysia | endemic | enzootic | unknown | unknown | unknown | (4) |
| CAAV | Alphavirus caaingua | Caainguá virus | South America | unknown | unknown | unknown | unknown | unknown | (5) |
| **CHIKV** | **Alphavirus chikungunya** | **chikungunya virus** | **Africa, Asia, Americas, Europe** | **epidemic** | **epizootic** | **arthritogenic** | **yes** | **symptomatic** | **(6)** |
| **EEEV** | **Alphavirus eastern** | **eastern equine encephalitis virus** | **North America, Caribbean** | **endemic** | **enzootic** | **encephalitic** | **yes** | **symptomatic** | **(7)** |
| EILV | Alphavirus eilat | Eilat virus | Middle East | unknown | unknown | unknown | no | unknown | (8) |
| FMV | Alphavirus fortmorgan | Fort Morgan virus | North America | endemic | enzootic | unknown | unknown | unknown | (9) |
| BUGCV | Alphavirus fortmorgan | Buggy Creek virus | North America | endemic | enzootic | unknown | unknown | unknown | (9) |
| GETV | Alphavirus getah | getah virus | Asia | endemic | enzootic | unknown | yes | asymptomatic | (10) |
| GETV;  SAGV | Alphavirus getah | getah virus;  Sagiyama virus | Asia | endemic | enzootic | unknown | yes | asymptomatic | (10) |
| GETV;  ALPHAV-M1 | Alphavirus getah | getah virus;  alphavirus M1 | Asia | endemic | enzootic | unknown | yes | asymptomatic | (10) |
| HJV | Alphavirus highlandsj | Highlands J virus | North America | endemic | enzootic | encephalitic | yes | asymptomatic | (11, 12) |
| MADV | Alphavirus madariaga | Madariaga virus | Central America, South America | endemic | epizootic | encephalitic | yes | symptomatic | (13) |
| **MAYV** | **Alphavirus mayaro** | **Mayaro virus** | **Central America, South America** | **epidemic** | **epizootic** | **arthritogenic** | **yes** | **symptomatic** | **(14, 15)** |
| MIDV | Alphavirus middelburg | Middelburg virus | Africa | endemic | enzootic | arthritogenic | yes | symptomatic | (16, 17) |
| NDUV | Alphavirus ndumu | Ndumu virus | Africa | endemic | enzootic | unknown | yes | unknown | (18) |
| **ONNV** | **Alphavirus onyong** | **o'nyong-nyong virus** | **Africa** | **epidemic** | **epizootic** | **arthritogenic** | **yes** | **symptomatic** | **(19, 20)** |
| ONNV;  IGORV | Alphavirus onyong | o'nyong-nyong virus;  Igbo Ora virus | Africa | epidemic | epizootic | arthritogenic | yes | symptomatic | (19, 20) |
| **RRV** | **Alphavirus rossriver** | **Ross River virus** | **Australia, Papua New Guinea, Pacific Islands** | **endemic** | **enzootic** | **arthritogenic** | **yes** | **symptomatic** | **(21, 22)** |
| SPDV;  SDV | Alphavirus salmon | salmon pancreas disease virus;  sleeping disease virus | Northern Atlantic, North Sea | endemic | enzootic | unknown | no | unknown | (23) |
| SESV | Alphavirus seal | southern elephant seal virus | Southern Ocean | endemic | enzootic | unknown | no | unknown | (24) |
| **SFV** | **Alphavirus semliki** | **Semliki Forest virus** | **Africa** | **endemic** | **enzootic** | **unknown** | **yes** | **symptomatic** | **(25)** |
| **SINV** | **Alphavirus sindbis** | **Sindbis virus** | **Europe, Asia, Africa, Australia** | **endemic** | **enzootic** | **arthritogenic** | **yes** | **symptomatic** | **(26)** |
| SINV;  OCKV | Alphavirus sindbis | Sindbis virus;  Ockelbo virus | Northern Europe | endemic | enzootic | arthritogenic | yes | symptomatic | (26) |
| SINV;  BABV | Alphavirus sindbis | Sindbis virus;  Babanki virus | Africa | endemic | enzootic | arthritogenic | yes | symptomatic | (26) |
| TROV | Alphavirus trocara | Trocara virus | South America | endemic | enzootic | unknown | unknown | unknown | (27) |
| **UNAV** | **Alphavirus una** | **Una virus** | **South America** | **endemic** | **enzootic** | **unknown** | **yes** | **unknown** | **(28, 29)** |
| **VEEV-IAB;**  **VEEV** | **Alphavirus venezuelan** | **Venezuelan equine encephalitis virus IAB;**  **Venezuelan equine encephalitis virus** | **Central America, South America** | **epidemic** | **epizootic** | **encephalitic** | **yes** | **symptomatic** | **(30-32)** |
| VEEV-IC;  VEEV | Alphavirus venezuelan | Venezuelan equine encephalitis virus IC;  Venezuelan equine encephalitis virus | Central America, South America | epidemic | epizootic | encephalitic | yes | symptomatic | (30-32) |
| VEEV-ID;  VEEV | Alphavirus venezuelan | Venezuelan equine encephalitis virus ID;  Venezuelan equine encephalitis virus | Central America, South America | endemic | enzootic | encephalitic | yes | symptomatic | (30-32) |
| VEEV-IE;  VEEV | Alphavirus venezuelan | Venezuelan equine encephalitis virus IE;  Venezuelan equine encephalitis virus | Central America, South America | endemic | enzootic | encephalitic | yes | symptomatic | (30-32) |
| VEEV-IF;  MDPV | Alphavirus mossopedras | Venezuelan equine encephalitis virus IF;  Mosso das Pedras virus | South America | endemic | enzootic | encephalitic | yes | symptomatic | (31-33) |
| VEEV-II;  EVEV | Alphavirus everglades | Venezuelan equine encephalitis virus II;  Everglades virus | North America | endemic | enzootic | encephalitic | yes | symptomatic | (34) |
| **VEEV-IIIA;**  **MUCV** | **Alphavirus mucambo** | **Venezuelan equine encephalitis virus IIIA;**  **Mucambo virus (Brazil, Suriname, Trinidad)** | **South America** | **endemic** | **enzootic** | **encephalitic** | **yes** | **symptomatic** | **(32)** |
| VEEV-IIIB  TONV | Alphavirus tonate | Venezuelan equine encephalitis virus IIIB;  Tonate virus | South America | endemic | enzootic | encephalitic | yes | symptomatic | (35) |
| VEEV-IIIC;  MUCV | Alphavirus mucambo | Venezuelan equine encephalitis virus IIIC;  Mucambo virus (Peru) | South America | endemic | enzootic | encephalitic | yes | symptomatic | (30-32, 36) |
| VEEV-IV;  PIXV | Alphavirus pixuna | Venezuelan equine encephalitis virus IV;  Pixuna virus | South America | endemic | enzootic | encephalitic | yes | symptomatic | (37, 38) |
| VEEV-V;  CABV | Alphavirus cabassou | Venezuelan equine encephalitis virus V;  Cabassou virus | South America | endemic | enzootic | encephalitic | yes | symptomatic | (39) |
| VEEV-VI;  RNV | Alphavirus negro | Venezuelan equine encephalitis virus VI;  Rio Negro virus | South America | endemic | enzootic | encephalitic | yes | symptomatic | (40-42) |
| WEEV | Alphavirus western | western equine encephalitis virus | North America; South America | endemic | enzootic | encephalitic | yes | symptomatic | (43) |
| WHATV | Alphavirus whataroa | Whataroa virus | New Zealand | endemic | enzootic | unknown | unknown | unknown | (44, 45) |

**Table S2.** Cryo-EM data collection, refinement, and validation statistics for VEEV VLPs treated with biFAB.

| Data set | VEEV 0.5:1.0 biFAB:Spike  (EMD-73769) | VEEV 1.0:1.0 biFAB:Spike  (EMD-73770) | VEEV 3.0:1.0 biFAB:Spike  (EMD-73781) |
| --- | --- | --- | --- |
| **Data collection and processing** |  |  |  |
| Magnification | 105,000 | 105,000 | 105,000 |
| Voltage (kV) | 300 | 300 | 300 |
| Electron exposure (e–/Å^2^) | 58 | 58 | 58 |
| Defocus range (μm) | -0.8 to -2.0 | -0.8 to -2.0 | -0.8 to -2.0 |
| Pixel size (Å) | 0.825 | 0.825 | 0.825 |
| Symmetry imposed | Icosahedral | Icosahedral | Icosahedral |
| Init. particle images (no.) | 124,203 | 48,882 | 6,888 |
| Final particle images (no.) | 91,421 | 41,959 | 6,539 |
| Map resolution (Å)  FSC threshold | 5.11  0.143 | 13.55  0.143 | 20.17  0.143 |

**Supplemental References:**

1.	A. L. P. Mosimann, M. K. de Siqueira, L. F. Ceole, C. Nunes Duarte Dos Santos, A new Aura virus isolate in Brazil shows segment duplication in the variable region of the nsP3 gene. *Parasit Vectors* **11**, 321 (2018).

2.	S. P. Jacups, P. I. Whelan, B. J. Currie, Ross River virus and Barmah Forest virus infections: a review of history, ecology, and predictive models, with implications for tropical northern Australia. *Vector Borne Zoonotic Dis* **8**, 283-297 (2008).

3.	C. M. Varghese, A. Milazzo, S. Hanson-Easey, Y. T. Damtew, P. Bi, The impact of temperature on Ross River virus and Barmah Forest virus infection in Australia: A scoping review. *Aust N Z J Public Health* **50**, 100291 (2026).

4.	Centers for Disease Control and Prevention (<https://wwwn.cdc.gov/arbocat/VirusDetails.aspx?ID=56>.

5.	M. K. Tscha *et al.*, Identification of a novel alphavirus related to the encephalitis complexes circulating in southern Brazil. *Emerg Microbes Infect* **8**, 920-933 (2019).

6.	A. A. Bettis *et al.*, The global epidemiology of chikungunya from 1999 to 2020: A systematic literature review to inform the development and introduction of vaccines. *PLoS Negl Trop Dis* **16**, e0010069 (2022).

7.	T. Corrin, R. Ackford, M. Mascarenhas, J. Greig, L. A. Waddell, Eastern Equine Encephalitis Virus: A Scoping Review of the Global Evidence. *Vector Borne Zoonotic Dis* **21**, 305-320 (2021).

8.	F. Nasar *et al.*, Eilat virus, a unique alphavirus with host range restricted to insects by RNA replication. *Proc Natl Acad Sci U S A* **109**, 14622-14627 (2012).

9.	C. H. Calisher *et al.*, Characterization of Fort Morgan virus, an alphavirus of the western equine encephalitis virus complex in an unusual ecosystem. *Am J Trop Med Hyg* **29**, 1428-1440 (1980).

10.	B. Li, H. Wang, G. Liang, Getah Virus (Alphavirus): An Emerging, Spreading Zoonotic Virus. *Pathogens* **11** (2022).

11.	M. C. Garvin *et al.*, Arboviral infection in two species of wild jays (Aves: Corvidae): evidence for population impacts. *J Med Entomol* **41**, 215-225 (2004).

12.	P. J. Meehan *et al.*, Epidemiological features of and public health response to a St. Louis encephalitis epidemic in Florida, 1990-1. *Epidemiol Infect* **125**, 181-188 (2000).

13.	J. Galue *et al.*, Contrasting Ecological Patterns of Venezuelan Equine Encephalitis and Madariaga Viruses in Small Mammal and Mosquito Populations from Two Enzootic Regions of Panama. *Vector Borne Zoonotic Dis* **25**, 749-760 (2025).

14.	M. C. Receveur, M. Grandadam, T. Pistone, D. Malvy, Infection with Mayaro virus in a French traveller returning from the Amazon region, Brazil, January, 2010. *Euro Surveill* **15** (2010).

15.	L. L. L. Wei, R. Tom, Y. C. Kim, Mayaro Virus: An Emerging Alphavirus in the Americas. *Viruses* **16** (2024).

16.	S. van Niekerk *et al.*, Sindbis and Middelburg Old World Alphaviruses Associated with Neurologic Disease in Horses, South Africa. *Emerg Infect Dis* **21**, 2225-2229 (2015).

17.	S. L. Graff *et al.*, Detection of two alphaviruses: Middelburg virus and Sindbis virus from enzootic amplification cycles in southwestern Uganda. *Front Microbiol* **15**, 1394661 (2024).

18.	R. H. Kokernot, B. M. McIntosh, C. B. Worth, Ndumu virus, a hitherto unknown agent, isolated from culicine mosouitoes collected in northern Natal. Union of South Africa. *Am J Trop Med Hyg* **10**, 383-386 (1961).

19.	G. Rezza, R. Chen, S. C. Weaver, O'nyong-nyong fever: a neglected mosquito-borne viral disease. *Pathog Glob Health* **111**, 271-275 (2017).

20.	K. D. Saxton-Shaw *et al.*, O'nyong nyong virus molecular determinants of unique vector specificity reside in non-structural protein 3. *PLoS Negl Trop Dis* **7**, e1931 (2013).

21.	D. Harley, A. Sleigh, S. Ritchie, Ross River virus transmission, infection, and disease: a cross-disciplinary review. *Clin Microbiol Rev* **14**, 909-932, table of contents (2001).

22.	B. Barber, J. T. Denholm, D. Spelman, Ross River virus. *Aust Fam Physician* **38**, 586-589 (2009).

23.	T. K. Herath *et al.*, Pathogenesis of experimental salmonid alphavirus infection in vivo: an ultrastructural insight. *Vet Res* **47**, 7 (2016).

24.	Z. E. Smeele, D. G. Ainley, A. Varsani, Viruses associated with Antarctic wildlife: From serology based detection to identification of genomes using high throughput sequencing. *Virus Res* **243**, 91-105 (2018).

25.	D. H. J. Cheong, B. Yi, Y. H. Wong, J. J. H. Chu, The Current Progress in the Quest for Vaccines Against the Semliki Forest Virus Complex. *Med Res Rev* **45**, 947-967 (2025).

26.	K. J. Sokoloski, D. Karki, C. M. Isom, S. Moni, The pathogenicity and virulence of Sindbis virus. *Virulence* **17**, 2609389 (2026).

27.	A. P. Travassos da Rosa *et al.*, Trocara virus: a newly recognized Alphavirus (Togaviridae) isolated from mosquitoes in the Amazon Basin. *Am J Trop Med Hyg* **64**, 93-97 (2001).

28.	L. A. Diaz, P. Diaz Mdel, W. R. Almiron, M. S. Contigiani, Infection by UNA virus (Alphavirus; Togaviridae) and risk factor analysis in black howler monkeys (Alouatta caraya) from Paraguay and Argentina. *Trans R Soc Trop Med Hyg* **101**, 1039-1041 (2007).

29.	T. M. Visser *et al.*, Effect of chikungunya, Mayaro and Una virus coinfection on vector competence of Aedes aegypti mosquitoes. *One Health* **20**, 100991 (2025).

30.	R. Tang *et al.*, Venezuelan Equine Encephalitis Virus: Structural Biology, Vaccines, and Advances in Functional Antibodies. *Vaccines (Basel)* **14** (2025).

31.	P. V. Aguilar *et al.*, Endemic Venezuelan equine encephalitis in the Americas: hidden under the dengue umbrella. *Future Virol* **6**, 721-740 (2011).

32.	N. L. Forrester *et al.*, Evolution and spread of Venezuelan equine encephalitis complex alphavirus in the Americas. *PLoS Negl Trop Dis* **11**, e0005693 (2017).

33.	M. B. Pisano *et al.*, Venezuelan equine encephalitis viruses (VEEV) in Argentina: serological evidence of human infection. *PLoS Negl Trop Dis* **7**, e2551 (2013).

34.	N. D. Burkett-Cadena, D. Fish, S. Weaver, A. Y. Vittor, Everglades virus: an underrecognized disease-causing subtype of Venezuelan equine encephalitis virus endemic to Florida, USA. *J Med Entomol* **60**, 1149-1164 (2023).

35.	I. Moltini-Conclois *et al.*, Vector competence of Aedes albopictus for Tonate virus highlights transmission risks in temperate and tropical regions. *Emerg Microbes Infect* **14**, 2547733 (2025).

36.	P. V. Aguilar *et al.*, Endemic Venezuelan equine encephalitis in northern Peru. *Emerg Infect Dis* **10**, 880-888 (2004).

37.	R. E. Shope, O. R. Causey, A. H. De Andrade, The Venezuelan Equine Encephalomyelitis Complex of Group a Arthropod-Borne Viruses, Including Mucambo and Pixuna from the Amazon Region of Brazil. *Am J Trop Med Hyg* **13**, 723-727 (1964).

38.	F. Cardozo *et al.*, Alphaviruses: Serological Evidence of Human Infection in Paraguay (2012-2013). *Vector Borne Zoonotic Dis* **18**, 266-272 (2018).

39.	R. M. Kinney, D. W. Trent, J. K. France, Comparative immunological and biochemical analyses of viruses in the Venezuelan equine encephalitis complex. *J Gen Virol* **64 (Pt 1)**, 135-147 (1983).

40.	S. R. Azar, R. K. Campos, N. A. Bergren, V. N. Camargos, S. L. Rossi, Epidemic Alphaviruses: Ecology, Emergence and Outbreaks. *Microorganisms* **8** (2020).

41.	M. B. Pisano *et al.*, First detection of Rio Negro virus (Venezuelan equine encephalitis complex subtype VI) in Cordoba, Argentina. *Mem Inst Oswaldo Cruz* **107**, 125-128 (2012).

42.	R. Loayza Mafayle *et al.*, Rio Negro Virus Infection, Bolivia, 2021. *Emerg Infect Dis* **29**, 1705-1708 (2023).

43.	L. Wang, R. Zheng, Z. Li, L. Zhang, Western equine encephalitis virus: A comprehensive review of epidemics, transmission, hosts, and strategies for mitigation. *Virulence* **16**, 2580162 (2025).

44.	J. A. Miles, The ecology of Whataroa virus, an alphavirus, in South Westland, New Zealand. *J Hyg (Lond)* **71**, 701-713 (1973).

45.	T. Maguire, J. A. Miles, J. Casals, Whataroa virus, a group A arbovirus isolated in South Westland, New Zealand. *Am J Trop Med Hyg* **16**, 371-373 (1967).
